# Metagenome of a polluted river reveals a reservoir of metabolic and antibiotic resistance genes

**DOI:** 10.1186/s40793-019-0345-3

**Published:** 2019-09-18

**Authors:** Parul Mittal, Vishnu Prasoodanan PK, Darshan B. Dhakan, Sanjiv Kumar, Vineet K. Sharma

**Affiliations:** 1 Metagenomics and Systems Biology Laboratory, Department of Biological Sciences, Indian Institute of Science Education and Research Bhopal, Madhya Pradesh, India; 2 Division of Glycoscience, School of Biotechnology, Albanova University Center, Royal Institute of Technology, 10691 Stockholm, Sweden

**Keywords:** Yamuna, Polluted river, Metagenomes, Antibiotic resistance genes, NDM-1, Metallo-β-lactamases, Metal tolerance

## Abstract

**Background:**

Yamuna, a major tributary of Ganga, which flows through the national capital region of Delhi, is among the major polluted rivers in India. The accumulation of various effluents, toxic chemicals, heavy metals, and increased organic load in the Yamuna directly affects the organisms that thrive inside or around this river. It also makes it an ideal site for studying the impact of pollution on the river microflora, which are sentinels of the water quality.

**Results:**

In this study, the microbial community structure and functional diversity of the Yamuna river water was assessed from the New Delhi region. The community structure of Yamuna during pre-monsoon (June) was found to be significantly different from the post-monsoon (November) time, with *Acinetobacter* being the most abundant genus during June, and *Aeromonas* during November. The functional characterization revealed the higher abundance of Methyl-accepting chemotaxis protein in the river water, which could be important for the microbial chemosensory adaptation in the environment. A higher abundance of genes related to nitrogen and sulfur metabolism, metal tolerance, and xenobiotic degradation, and complete degradation pathways of aromatic compounds such as toluene, xylene, benzene and phenol were identified. Further, the results showed the presence of a pool of antibiotic resistance genes in the bacterial microbiome in the Yamuna alongside a large number of broad-spectrum antibiotics, such as carbapenemases and metallo-β-lactamases. Efflux mechanism of resistance was found to dominate among these microbes conferring multi-drug resistance. The Principal Coordinate Analysis of the taxonomic composition of the Yamuna River water with publicly available freshwater and sewage datasets revealed significant differences in the two Yamuna samples and a greater resemblance of pre-monsoon Yamuna sample to sewage sample owing to the higher pollution levels in Yamuna in the pre-monsoon time.

**Conclusion:**

The metagenomic study of the Yamuna river provides the first insights on the bacterial microbiome composition of this large polluted river, and also helps to understand the dynamics in the community structure and functions due to seasonal variations. The presence of antibiotic resistance genes and functional insights on the metabolic potential of a polluted river microbiome are likely to have several applications in health, biotechnology and bioremediation.

## Background

With the rapid growth in human population, industrialization, and urbanization, the pollution levels in rivers have increased drastically. The freshwater is required to meet the demands of the human population; however, the dumping of domestic, industrial and agricultural wastes into the freshwater sources have led to its rapid deterioration. A wide variety of untreated organic and inorganic pollutants, including fecal wastes, industrial effluents, oils, grease, plastics, plasticizers, aromatics, pesticides and heavy metals are discharged into the rivers. Resultantly, many rivers have been converted into sewage carrying drains, which pose an immense threat to the ecosystem. A similar scenario exists in India, where several major rivers show high levels of pollution affecting the human population and the surrounding ecosystem [1–5].

The Yamuna, the longest tributary of river Ganga, is among the most polluted rivers in India [6, 7]. It originates from the Yamunotri glacier, flows through 1376 km before merging into the Ganges at Allahabad. Yamuna receives outfalls from 18 major drains in the Delhi region (Central Pollution Control Board (CPCB) 2015). The untreated discharge of urban runoffs consisting of fecal waste, hospital waste, and other domestic waste, and industrial effluents are the major contributors of pollution, causing an increase in the organic load, toxic chemicals, and heavy metals in the river [8, 9]. According to water assessment reports of Yamuna, 0.1–1.1 mg/l DO, 29–67 mg/l BOD and 230,000–160,000,000 MPN/100 ml coliform content were observed in 2016 at a site in New Delhi (CPCB 2017). The low levels of dissolved oxygen and very high levels of BOD are indicators of deteriorating quality of the river water.

Microbes are essential components of aquatic ecosystems and possess a vast array of metabolic genes and are the major agents of biogeochemical cycling [10]. However, the bacterial communities in a polluted river like Yamuna thrive on the accumulated organic load, toxic chemicals, xenobiotics and heavy metals present in the river. In such an environment, the bacterial microbiome is expected to possess genes capable of degrading various pollutants, including organic compounds, toxicants, and xenobiotics. Further, the urban discharge also leads to an accumulation of antibiotics in the receiving drains that merges into the Yamuna River [11–15]. Antibiotics such as Ampicillin, Ciprofloxacin, Gatifloxacin, Sparfloxacin, and Cepuroxime have been detected in the Yamuna river at different sites in the New Delhi region [15]. The detection of antibiotics and discharge of a large number of sewage drains into the river hints towards the presence of a pool of resistome residing in the Yamuna [16]. However, only a little is known about the prevalence of ARGs in the river, which is a major source of water for a large population in India.

Understanding the dynamics in community structure and function across contaminated freshwater sources, such as the Yamuna, helps in determining the impact of human practices on the water ecosystems. The unique environmental characteristics and the presence of eutrophication of the Yamuna river makes it a distinct study site for exploring the bacterial community structure, which is poorly characterized for this river. Thus, the present work identifies the bacterial communities present in the Yamuna River water using metagenomic approaches. The pollution levels in Yamuna shows drastic variations between the pre-monsoon and post-monsoon time. Therefore, to capture the bacterial diversity of the river and to understand the differences between the two seasons, the metagenomic assessments were carried at two time points: June (pre-monsoon) and November (post-monsoon). This is the first study to provide the glimpses into the functional characteristics along with bacterial diversity of the microbiome from the Yamuna river. Since this river is a freshwater source, which is being contaminated with sewage water, a comparative analysis of the Yamuna river metagenome with sewage and freshwater metagenomes were also performed.

## Results

### Taxonomic analysis

**Fig. 1:**
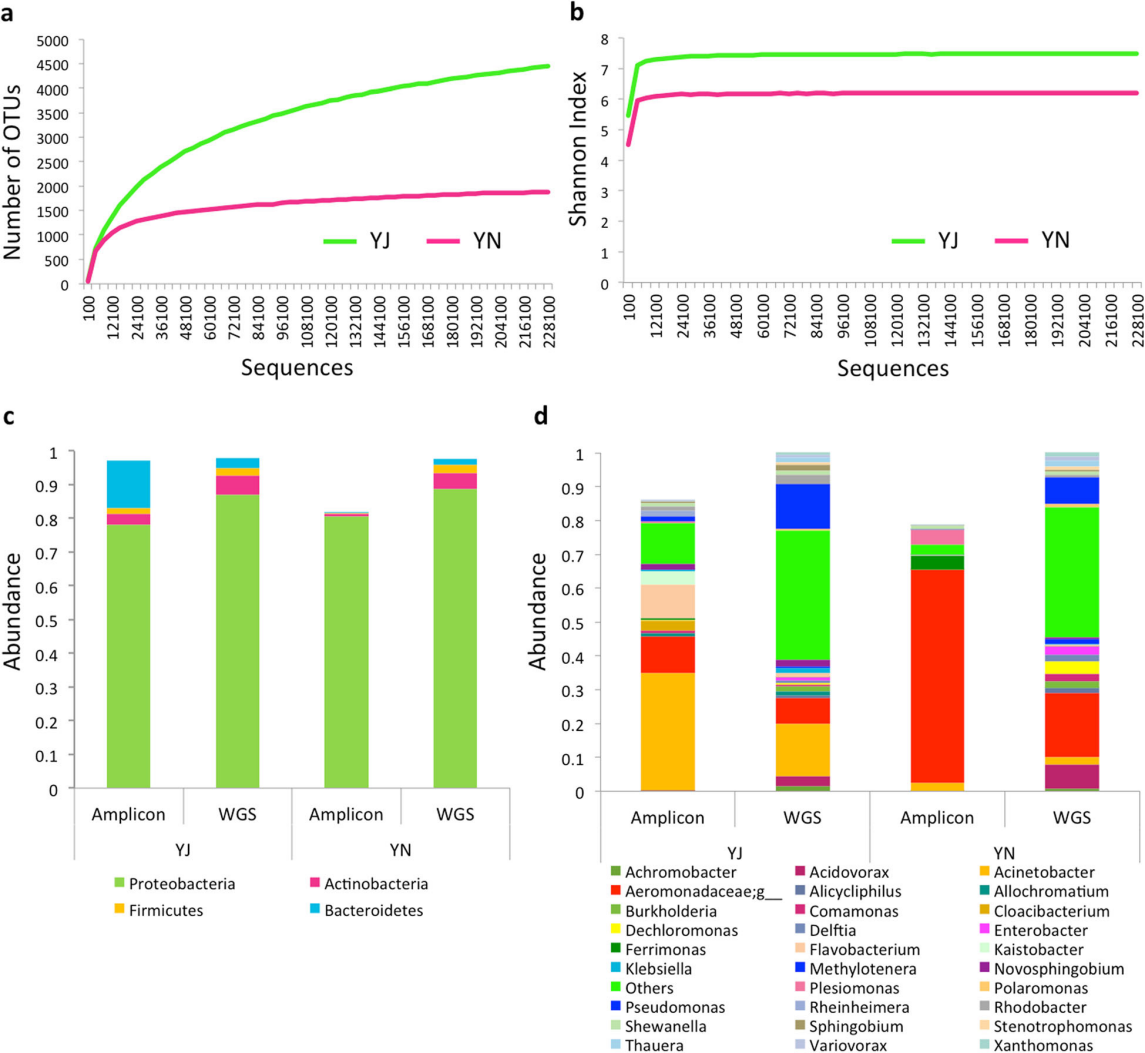
Microbial composition in the two Yamuna water samples. The bacterial alpha diversity in June (YJ) and November (YN) water samples: **a** Number of OTUs and **b** Shannon index. **c** The distribution of major contributing phyla (having more than 1% abundance). **d** The distribution of major contributing genus (having more than 1% abundance) in the two samples

Taxonomic assignment was performed for the V3 hypervariable region of 16S rRNA using QIIME to examine and compare the composition of bacterial community in samples collected at two different time points, June (YJ) and November (YN). A total of 250,904 and 167,020 OTUs were obtained after clustering of 7,451,906 and 1,596,945 high-quality reads from YJ and YN samples, respectively (Additional file 1: Table S1). The estimates of alpha diversity indices showed higher phylogenetic diversity and evenness in YJ as compared to YN (Fig. 1a, b). The observed number of OTUs and Shannon index were higher in the case of YJ sample.

#### Taxonomic analysis using amplicon reads

The bacterial community structure of YJ and YN was determined by the taxonomic assignment of amplicons (OTUs) using the Greengenes database. At the phylum level, both samples consisted of Proteobacteria (78% in YJ and 80% in YN) as the most abundant phylum (Fig. 1c). However, differences were observed in the relative proportions of the other phyla. YN showed a higher proportion of unassigned phyla (18%), whereas, YJ showed a higher proportion of Bacteroides (14%) and Actinobacteria (3%). The community structure observed at the family and genus level showed major differences in abundance between the two samples (Fig. 1d and Additional file 1: Figure S1). *Acinetobacter*, a Gammaproteobacteria, was found to be the most abundant genus in YJ (35%), whereas, unknown genera from family Aeromonadaceae were found to the most abundant in YN (48%).

To identify the most represented genus in the YN sample, we identified the top ten OTUs with the highest number of sequences. A total of eight OTUs (57. 4% of total sequences), out of these ten most abundant OTUs, belonged to unknown genera from the family Aeromonadaceae in YN sample. Interestingly, we found that all these OTUs belonged to genus *Aeromonas* after aligning them against NT database at NCBI web portal using blastn, and showed the top hit to species *Aeromonas hydrophila* (Additional file 1: Table S2). In YJ, the most abundant OTU (8. 6% of total sequences) was assigned as *Flavobacterium*, whereas, four out of the ten most abundant OTUs belonged to genus *Acinetobacter*. Of these, one OTU was assigned to species *Acinetobacter johnsonii*, covering 4% of total sequences (Additional file 1: Table S3). The presence and abundance of *Acinetobacter* and *Aeromonas* in polluted water and sewage sites have been reported in several studies [17]. Both these genera are also known to harbor antibiotic resistance genes [18] in sewage such as in wastewater treatment plants, hospital and pharmaceutical sewage.

#### Taxonomic classification using WGS metagenomic data

The whole genome shotgun (WGS) sequencing reads were generated on Illumina HiSeq platform, and a total of 25,877,683 and 87,301,705 high quality reads for YJ and YN, respectively, were obtained after quality filtration steps. These WGS reads were subjected to taxonomic classification using Kraken [19] at the highest possible taxonomic levels. We observed that a large percentage of reads (55% in YJ and 61% in YN) could not be assigned any taxonomy. Among the assigned ones, a majority of the reads, 87 and 89% in YJ and YN, respectively, belonged to Proteobacteria. These results corroborate well with the high abundance (78 and 80% in YJ and YN, respectively) of Proteobacteria observed from the analysis of amplicon reads (Fig. 1c). In YJ, the genus *Acinetobacter* was observed as the most abundant genus using both WGS (15.5%) and amplicon (34.8%) datasets (Fig. 1d), whereas, unassigned genus belonging Aeromonadaceae family was the most abundant in both amplicon (62.8%) and WGS (18.8%) in YN. Altogether, the taxonomic classification of WGS sequences also support the differences in the taxonomic composition in the two Yamuna samples, and the higher abundance of *Acinetobacter* in YJ and Aeromonadaceae genus in YN sample.

### Functional analysis

A total of 796,860 and 1,567,548 contigs were generated from YJ and YN datasets, respectively. From these contigs, a total of 962,761 and 1,776,601 ORFs were identified in YJ and YN, respectively. A total of 710,715 and 1,332,740 ORFs from YJ and YN datasets could be mapped to the KEGG database, and a total of 9152 KOs and 2661 ECs were identified in YJ and YN samples belonging to 1344 different KEGG Pathways. Methyl-accepting chemotaxis protein (MCP) was observed as the most abundant KO in YN sample (0. 34%) and was also among the five most abundant KOs in YJ (Additional file 2: Table S4). MCPs are transmembrane receptors that sense the concentration of attractants and repellants and mediate chemotaxis. The pathways related to ABC transporters (3.5%), two-component system (3.2%), amino acid biosynthesis (2.5%), and carbon metabolism (1.5%) were among the most abundant pathways in the Yamuna (Additional file 2: Table S5). An abundance of Nitrogen (0.8%) and Sulfur (0.8%) metabolism pathways were also observed in the Yamuna water microbiome. The presence of complete pathways for nitrogen and sulphur metabolism and aromatic compound degradation such as toluene, xylene, benzene and phenol were also found in the samples. Further, a large number of genes related to antibiotic resistance and metal tolerance were observed. A detailed analysis of β-lactam resistance pathway was carried out that revealed antibiotic-resistance mechanisms present in bacteria in a polluted river. It showed the presence of genes for penicillin binding, inhibition of peptidoglycan biosynthesis, β-lactamase induction by muropeptides via AmpG-AmpR-AmpC and Opp-BlaI-BlaZ pathways leading to hydrolytic degradation, and also possesses RND efflux pumps for efflux of the β-lactams (Additional file 2: Table S5).

#### Antibiotic resistance genes

The antibiotic resistance genes (ARGs) were identified in YJ and YN samples using the CARD database [20], which consists of 3008 sequences classified into 40 categories. Only those genes predicted from the metagenomic reads, which was confirmed from the assembled contigs were considered for the analysis. Both YJ and YN samples showed an almost similar abundance of different categories of ARGs, and hence were clubbed and discussed together as a single set called ‘YARG’ in the subsequent section. A list of the identified ARGs in YJ and YN is provided in Additional file 2: Table S6a, b. From the assembled data including both YJ and YN datasets, a total of 662 subtypes of ARGs (538 genes) belonging to 34 CARD categories were found in YARG. rpoB gene, which encodes the beta subunit of RNA polymerase and provides resistance to rifampicin [21], was found to be the most abundant (9–10%) in the YARG. It was followed by rpoC (~ 7%), which also encodes the beta subunit of RNA polymerase and provide resistance to daptomycin [22]. The gyrA gene, which encodes DNA gyrase and is responsible for providing resistance to fluoroquinolones [23], was also abundant (~ 5%). Similarly, adeJ gene, which encodes the multiple efflux protein AdeJ [24], was found abundant (1–4%) in YARG; however, it showed a higher abundance in YJ dataset. This gene is known to be present in *Acinetobacter* [24], which was also the most abundant genus observed in the YJ sample.

Among the 40 categories, the most abundant gene categories found in YARG were the antibiotic resistance gene variant or mutant (20.2%), fluoroquinolone resistance gene (11. 7%), and efflux pump conferring antibiotic resistance (13.4%). All the genes, which are known to confer aminocoumarin resistance, were found in YARG (Additional file 2: Table S7). The genes conferring resistance to rifampin, macrolide, chloramphenicol, tetracycline, phenicol, aminocoumarin, β-lactams, lipopeptides, elfamycin, polymyxins, aminoglycosides, isoniazid, trimethoprim, lincosamide were found in the Yamuna.

MacA-MacB and MtrC-MtrD-MtrE are two important and well-studied Macrolide resistance efflux systems [25, 26]. All the genes involved in MtrC-MtrD-MtrE efflux system, and MacA, which is a part of MacA-MacB efflux system, could be identified in YARG, whereas, MacB could not be identified as it was absent in the reference database. A total of 51 genes responsible for multidrug resistance were found in YARG. All these multidrug resistance genes use an efflux system for resistance. Interestingly, a large number of ARGs were involved in the efflux system (13% in YJ; 16% in YN) in the Yamuna metagenome (Additional file 2: Table S6 and S7) suggesting that the microbial communities have acquired resistance mainly through the efflux mechanisms.

A total of 164 genes encoding β-lactamases were found in YARG, consisting of Class A including CARBs and Tla, Class B including cephA3 and cphA6, Class C including CMY, MIR, PDC, DHA, and OCH, and Class D including OXA β-lactamases. A large number of carbapenemase type β-lactamases (such as IMP, VIM and OXA) were identified in the Yamuna, of which eight were metallo- β-lactamases (Additional file 2: Table S6). These include bla_NDM-1_, bla_NDM-8_, bla_AIM-1_, SMB-1, bla_IMP-1_, bla_IMP-25_, imiH, and bla_VIM-2_. Interestingly, among the different Metallo-β-lactamases, bla_NDM-1_and bla_NDM-8_, which encode New Delhi-Metallo-β-lactamase-1 (NDM-1) and New Delhi-Metallo-β-lactamase-8 (NDM-8) were also identified. NDM has gained much attention recently due to their broad-spectrum resistance to antibiotic, including cephalosporins, moxalactam, and carbapenems and has been identified in North Indian river microbiomes recently [27].

#### Genes for xenobiotic degradation

Due to the higher abundance of xenobiotic compounds in the river, the microbes tend to acquire genes and pathways for transformation or transportation of these chemicals as a part of the survival mechanism. We examined the genes responsible for such biotransformations in the Yamuna river microbiome. The complete metabolic pathways for Azathioprine, its pro-drug 6-Mercaptopurine, Capecitanine and Irinotecan were observed in Yamuna waters from the KEGG analysis. In total, 131 enzymes capable of acting on different drugs were identified in Yamuna out of 370 enzymes in the Drugbank database. These enzymes are involved in the biotransformation and transportation of drug. Several enzymes responsible for multiple effluxes of drug molecules were also identified such as Multidrug resistance protein 1, multidrug resistance-associated protein-4, 5, 6 and 7. These observations point towards the prevalence of multidrug resistance though efflux systems in the Yamuna River microbial communities. The xenobiotic degradation by microbes may potentially affect the toxicity and efficacy of drugs with respect to human health [28].

#### Metal tolerance

Heavy metal contamination in the Yamuna river can severely affect the river microflora, and thus the microbes acquire metal tolerance for their survival in the environment [29]. We, therefore, investigated the metal tolerance in the microbes. Genes related to metal tolerance in the assembled contigs of YN and YJ were identified using BacMet database [30], a manually curated database consisting of 444 sequences for metal resistance. Out of 335 genes classified into 72 categories, 271 metal tolerant genes belonging to 47 different categories were identified in the Yamuna (Additional file 2: Table S8). According to tolerance to different compounds/elements, Cu, Ni and Zn were found to have a maximum number of metal tolerant genes in the Yamuna. These are trace elements and are required by microbes. Thus, the identification of tolerant genes for these elements was expected. Interestingly, a higher number of genes were involved in tolerance or biotransformation to heavy metals such as Hg, Co and Ar. A total of 47 and 46 unique genes were found to confer tolerance to Hg and Co, respectively (Additional file 2: Table S8). Hg resistance is mainly acquired by ‘mer’ operon. Out of the 17 known mer genes (present in BacMet database), 13 genes were identified in the river microbiome. The genes involved in Arsenic resistance (ars genes), namely *arsR*, *arsA*, *arsB*, *arsC*, *arsD*, *arsH* and *arsM* were also identified in the Yamuna River (Additional file 2: Table S8).

### Comparative analysis

To understand the differences in the bacterial microbiome composition in two seasons datasets and to compare the microbiome of Yamuna waters, we performed a detailed comparative analysis with sewage and freshwater samples. Alpha diversity of YJ, YN, sewage (SW), and freshwater (FA and FN) datasets were examined using Shannon index and number of OTUs (Observed Species). With respect to the number of observed species, YJ and YN were found to be more diverse as compared to sewage and freshwater (Additional file 1: Figure S2). The sewage sample showed higher Shannon index denoting higher evenness in the sample. Overall, the alpha diversity was inconsistent for the two Yamuna samples and consistent for the two freshwater samples, and the diversity for Yamuna samples differed with both sewage and freshwater samples.

**Fig. 2:**
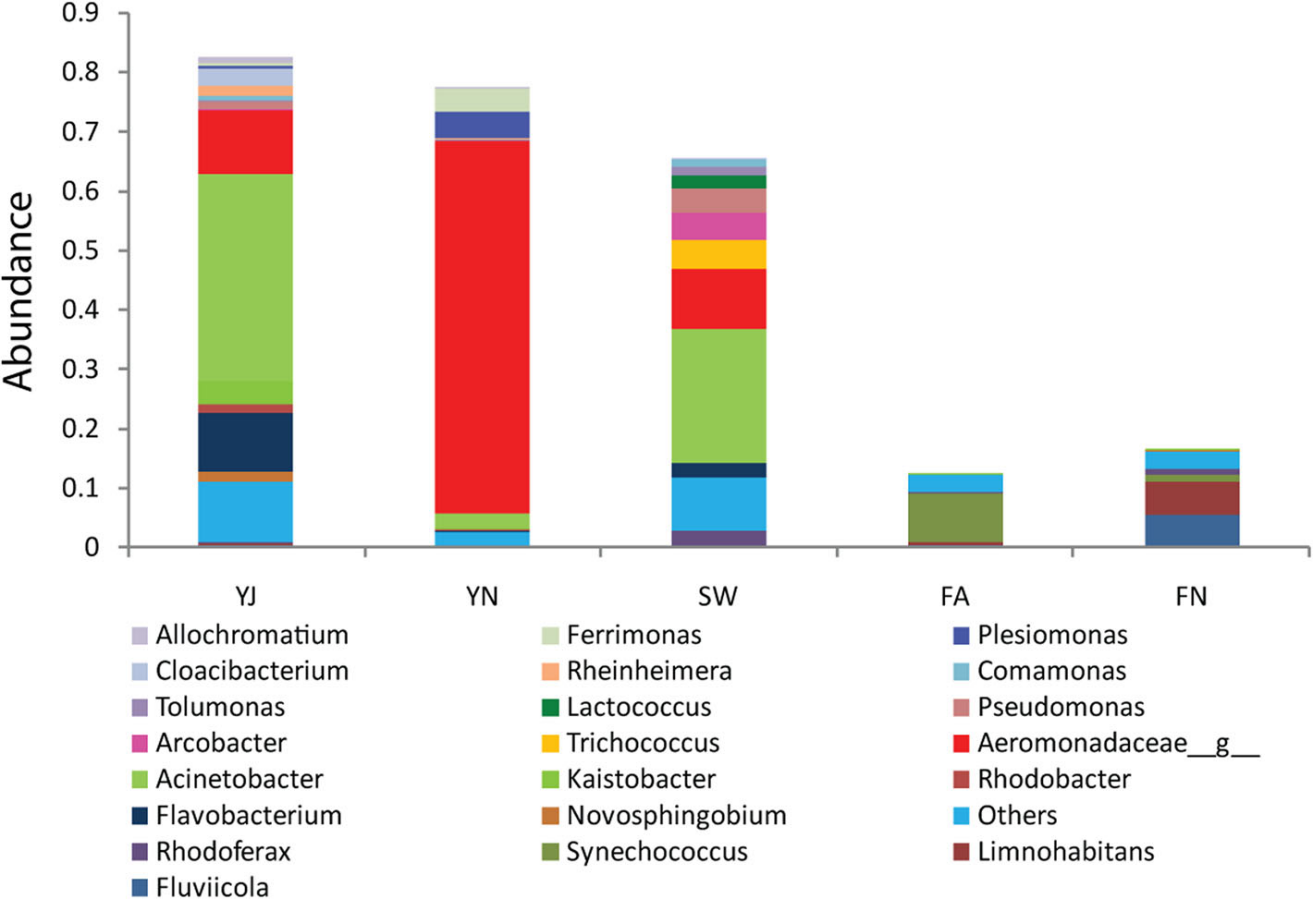
Microbial distribution among the five datasets. The Genus level composition having more than 1% abundance in the five datasets are shown

The taxonomic structure of the five datasets at the phylum level (Additional file 1: Figure S4) indicates that the phylum Proteobacteria was the most dominant phyla in both sewage (60%) and Yamuna samples (~ 80%), whereas, Proteobacteria constituted only ~ 36% in the case of freshwater samples. It is apparent that the microbial composition of YJ and YN showed more similarity to sewage as compared to the freshwater. Actinobacteria was dominant in both the Freshwater samples (~ 40%) and was less abundant in the other three datasets (< 4%). Similarly, at the family level, YJ and YN displayed more similarity with sewage. The family Moraxellaceaea was the most abundant family in sewage (23. 9%) and YJ (37. 5%), whereas, Freshwater (0%) and YN (2. 5%) showed less representation of this family (Additional file 1: Figure S5). Notably, the freshwater samples showed a very different taxonomic composition in comparison to the sewage and Yamuna water samples (Fig. 2). At the genus level, YJ and were dominated by genus *Acinetobacter* (34 and 22%, respectively), whereas, YN was dominated by unassigned genus from family Aeromonadaceae (64%)**.**

**Fig. 3:**
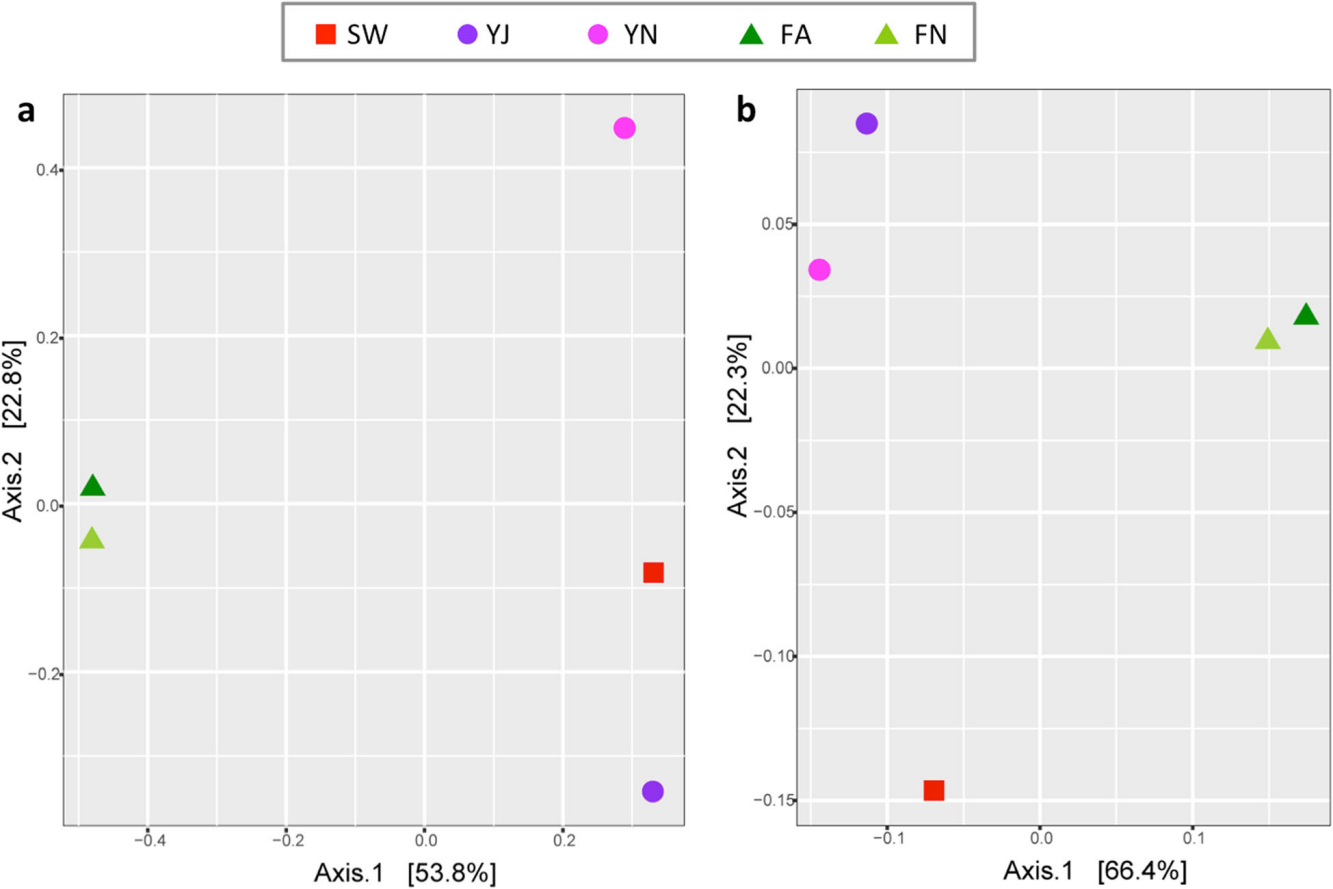
Principle Coordinates Analysis (PCoA) of pairwise dissimilarities (Bray-Curtis distances) among the five datasets. The distances are based on **a** community composition from amplicon analysis and **b** Functional composition using KEGG database

The inter-sample diversity estimated using ordinations of the Bray-Curtis distance shows that YJ and YN samples are distant from each other and also distant from freshwater samples in terms of the microbial communities in the Principal Coordinate Analysis (Fig. 3a). The observed distance between the YJ and YN samples indicates higher seasonal variations in microbial diversity in the Yamuna. YJ was found closer to Sewage sample in terms of microbial communities, which corroborates with the previous reports suggesting that the pollution level in the Yamuna river is at the peak during summers (May–June) and the scenario changes after the beginning of the monsoon [31]. Contrarily, the PCoA analysis carried out using the functional profile (KEGG and eggNOG database) showed that both the Yamuna samples were close to each other, and all three types of datasets, Yamuna, freshwater and sewage appeared distant from each other (Fig. 3b and Additional file 1: Figure S3). This observation suggests that in comparison to the large differences observed in the taxonomic composition of the two seasons, the differences were lower at the functional level. More studies in the future with more number of datasets and time-points will provide further insights into the seasonal differences in Yamuna microbiome.

**Fig. 4:**
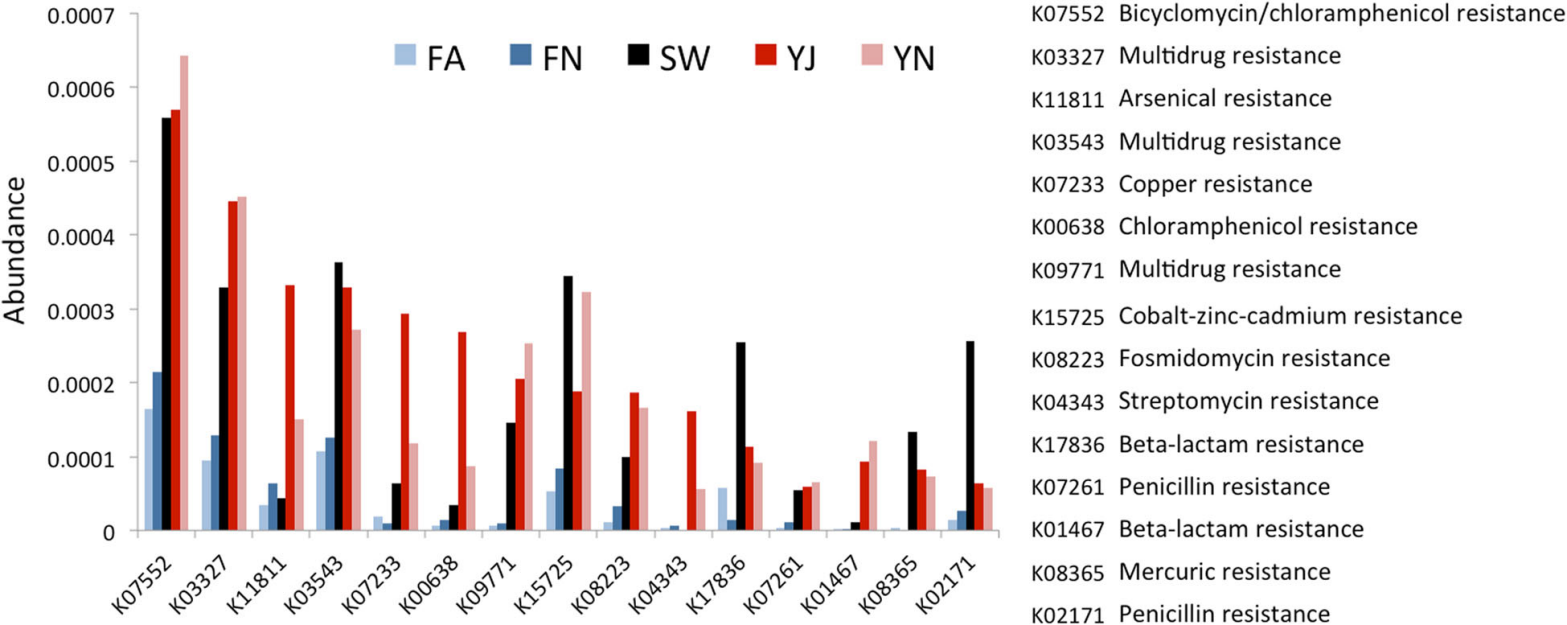
KOs related to metal tolerance and antibiotic resistance in the five datasets

**Fig. 5:**
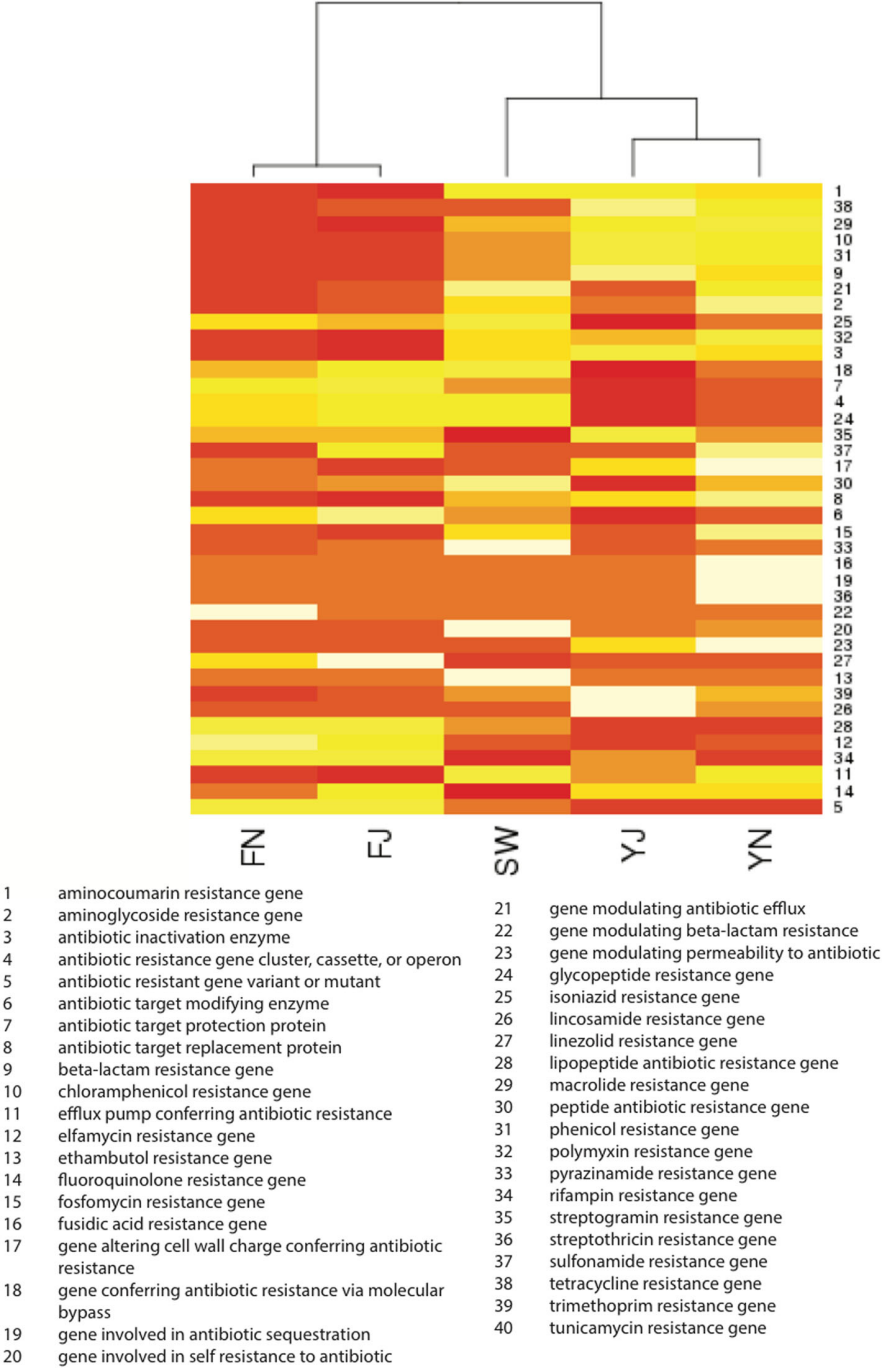
Heatmap showing abundance of antibiotic resistance gene categories in the five datasets

A comparison in the abundance of KEGG Orthologs related to metal tolerance and antibiotic resistance was carried out using the five datasets, which showed that the two Yamuna samples showed similar profiles with each other and with the Sewage for most KOs (Fig. 4). The antibiotic resistance genes categories among the five datasets were visualized on a heatmap, which showed a clustering of the freshwater samples. The two Yamuna samples also clustered together and were closer to sewage compared to freshwater on the heatmap (Fig. 5). The KEGG pathway-based comparison of YJ and YN with freshwater revealed that ChpA-ChpB/PilGH (chemosensory) and EnvZ-OmpR (osmotic stress response) two-component regulatory systems, assimilatory sulfate and nitrate reduction pathways were significantly associated with the Yamuna (*p*-value < 0.05), whereas, amino acid biosynthesis pathways and nucleotide sugar biosynthesis pathways were associated with freshwater (*p*-value< 0.05; Additional file 2: Table S9). These results can be attributed to the availability of high amounts of organic matter for bacterial community thriving in sewage and Yamuna.

## Discussion

The river Yamuna faces extremes of dry and flood-like conditions in a year. During summers (May-Jun), the river is almost in a dry state and mainly contains the outfalls of various drains [32]. These outfalls carry untreated or partially treated domestic and industrial wastewater. The river gets recharged during monsoon (Jul-Oct), and thus, it shows an improved water quality during the post-monsoon season (Oct-Nov). To gain insights into the bacterial composition in Yamuna at both taxonomic and functional levels, we chose two time points June and November, which shows two entirely different conditions of the river. The taxonomic analysis highlights the differences in the microbial community structure between the two time points and revealed that taxonomic diversity is higher during June than in November. It also unveiled that *Acinetobacter* (in June) and *Aeromonas* (in November), belonging to the same taxonomic class, are the most abundant genera in the Yamuna. The comparative analysis with freshwater and sewage samples shows that the taxonomic composition in YJ and YN are entirely different, and the diversity in YJ is closer to sewage than YN. This corroborates with the dry state of Yamuna in summers containing mainly the wastewater.

Recent studies suggest that the urban effluents contribute to antibiotic pollution in the receiving drains and water bodies [33]. The extensive use of antibiotics in the form of medication leads to the development of antibiotic resistance in microbes residing in the human body [34]. Through feces, these antibiotics and the resistant microbes become common component of the waste streams and contaminate the environment [18, 35]. A recent study estimated that 53,800 tons of antibiotics were released to open environment, majorly entering into rivers, following wastewaters in China in 2013 [36]. India is among the largest consumers of antibiotics and the Yamuna receives domestic and industrial waste from one of the most populated cities [37]*.* Thus, several classes of antibiotics persists in the river [15], which is likely to contribute to the development and maintenance of antibiotic resistance in microbes. Previous studies have also shown that few species of *Acinetobacter* and *Aeromonas*, the two most abundant genera identified in the Yamuna, also harbors various antibiotic-resistance genes [38–40].

The functional analysis using the CARD database shows that the microbial community residing in the Yamuna possesses a large number of antibiotic resistance genes. Most of the YARGs mainly belong to efflux systems, showing resistance to multiple antibiotics. Several Metallo-β-lactamases, that catalyze the hydrolysis of most β-lactams antibiotics genes were identified in the river water. Notably, the NDM genes (bla_NDM-1_ and bla_NDM-8_) were also identified in our analysis, which makes the carrying bacterium the most resistant strain till date. bla_NDM-1_ was first reported in 2008 in *Klebsiella pneumoniae* isolated from a Swedish patient repatriated after admission to hospital in New Delhi [41]. Since then, the gene and its several variants have been identified in several hospitals worldwide. The presence of a large number of ARGs in Yamuna could be a consequence of the disposal of domestic and hospital wastes into the river. Several studies have reported that antibiotics and antibiotic resistance genes in the freshwater sources are mainly derived from pollution with human or animal waste [36, 42–44]. The Yamuna water is main source of water in several regions in north-India, and is currently used for in-stream purposes such as bathing, clothes washing and cattle wading. The presence of antibiotic resistance genes in microbes in such potable water sources poses a potential health hazard.

The functional analysis shows that chemotactic proteins (MCP), and pathways related to chemosensory two-component regulatory systems were abundant in the Yamuna, which suggests the substantial role of chemosensory motility system in these microbes. Chemotaxis helps bacteria to find optimum conditions for their growth and survival. They migrate to patches of enriched nutrients and away from toxins through concentration gradients. There are recent discoveries in bacterial chemotaxis toward pollutants, and its application in bioremediation [45]. A large number of genes conferring resistance to heavy metals, such as Hg, Co and Ar, were identified in this study. The metal tolerant bacteria have also been previously identified in the Yamuna River [46, 47]. For years, Yamuna received untreated discharges from industrial effluents, contaminated by heavy metals. Moreover, the immersion of painted idols directly into the river also contributes to heavy metal contamination (CPCB 2018). Due to their accumulation and non-degradable nature, the concentration of heavy metals in Yamuna, including Ar, Cr, Fe, Ni, Cu, Pb, and Hg exceeds the standard maximum permissible limit [7, 48]. The metal tolerance genes and microbes identified in the river could be an important resource for decontamination of the environment and have potential applications in bioremediation [49]. Further, the identification of complete degradation pathways of aromatic compounds such as toluene, xylene, benzene and phenol in the samples indicate the potential of such ecosystems in discovering novel enzymes and species in further studies which may find important applications in bioremediation and biotechnology.

## Methods

### Sample collection and DNA extraction

The river water samples were collected in duplicate in sterile plastic bottles from two different locations (28.627552 N, 77.253977 E) at a distance of about 15 m from the banks and one meter depth from the surface from the Yamuna (YAM) River, near ITO Bridge, New Delhi, India. The samples were collected at two different time points i.e. in the month of June (YJ) and November (YN) and were transported to the laboratory at 4 °C and stored at − 20 °C until further processing. Each sample was filtered through 1.2 μm pore size membrane to remove debris and coarse particles, further passed through a 0.2 μm pore size to collect the prokaryotic cells on the filter. Extraction of metagenomic DNA was performed in less than a week of sample collection as per the manufacturer’s instructions using Metagenomic DNA Isolation Kit for water (Epicentre).

### 16S rRNA amplicon sequencing and analysis

The 16S rRNA V3 region was amplified from the Yamuna November (YN) and June (YJ) DNA samples using the general primer pairs 341F - CCTACGGGAGGCAGCAG and 534R – ATTACCGCGGCTGCTGGC [50]. The amplified products were further extracted using QIAquick Gel Extraction Kit (QIAGEN) and used for sequencing. The purified 16S rRNA V3 amplicons were sequenced using Illumina HiSeq sequencing platform, which generated a total of 13,565,755 and 191,740,397 paired-end reads for YJ and YN samples, respectively. The reads were filtered by removing unambiguous bases using the NGS QC Toolkit v2.3.3 [51] and were merged into single reads using FLASH [52]. The low quality reads were removed, and the primers were trimmed from both the ends using Cutadapt v1.8.3 [53], to obtain high quality 16S rRNA V3 sequences for YN and YJ samples, respectively.

OTUs were picked from the filtered reads using closed-reference OTU picking from QIIME v1.9 at 97% identity against the Greengenes database (v13_5) [54]. The reads that failed to cluster using closed reference OTU picking were clustered using de novo OTU picking. The representative sequences were extracted from OTUs and aligned against the Greengenes database using BLAT. The hits, which showed an identity ≥90% and aligned length ≥ 100 bp, were selected and the taxonomy was assigned by the ‘Lowest Common Ancestor’ approach using in-house Perl scripts. The samples were rarefied 10-times from 100 sequences with a step size of 4000. The diversity metrics, namely ‘Observed species’ and ‘Shannon diversity index’ were calculated at each rarefied depth to estimate the intra-sample diversity.

### Metagenome sequencing and analysis

The YJ and YN samples were sequenced using Illumina sequencing HiSeq platform, generating a total of 96,000,349 and 165,873,760 paired-end reads, respectively. The reads containing unambiguous bases and the low-quality reads were removed using the NGS QC Toolkit [51]. The paired-end reads were assembled into single reads using FLASH [52] resulting in a total of 25,877,683 and 87,301,705 high quality reads for YJ and YN, respectively. The reads were taxonomically classified using Kraken v0.10.5 [19] to study the microbial community structure.

Several publicly available assembly tools including MetaVelvet, SOAPdenovo, MegaHit and Genovo were evaluated at different k-mers to assemble the reads. MEGAHIT v1.1.1 [55] displayed the best (N50 value 492) performance among these methods and was used to assemble the short Illumina reads into contigs using the default parameters. The analysis of metagenomic data was carried out for both reads and assembled contigs. For the reads-based analysis, the paired-end reads generated for YJ and YN were combined into single reads using FLASH, and ORFs were predicted in the high-quality reads using MetaGeneMark (v3.25) [56], and the functional annotation was carried out using RAPsearch [57] against KEGG and EggNOG databases v4.5.1 [58] with a maximum e-value cut-off of 10^− 6^ and aligned length ≥ 30 amino acids. Antibiotic resistance genes were identified by aligning the ORFs against CARD database v1.1.7 [20] using RAPsearch with an e-value cut-off of 10^− 6^ and aligned length ≥ 30 amino acids. ORFs from the contigs were predicted using MetaGeneMark and were searched against CARD [20], BacMet v1.1 [30], and DrugBank v5.0 [59] databases for functional annotation using RAPsearch with threshold parameters of the minimum aligned length of 50 amino acids or at least 50% query coverage, and E-value ≤10^− 6^.

### Comparative analysis

To compare the microbial diversity and gene pool of the Yamuna river samples (YN and YJ) collected with other related metagenomes, amplicon and whole-genome sequencing (WGS) datasets from a freshwater and a sewage site were retrieved. The freshwater dataset was obtained from Lake Lanier generated for the month of August (FA) and November (FN) [60], which consists of 235,469 and 256,503 amplicon reads from the V1–3 16S rRNA region and a total of 61,659,612 and 34,204,450 WGS reads from FA and FN, respectively, generated using the 454 platform. The sewage dataset (SW) was obtained from Jones Island and South Shore [61] containing a total of 237,559 amplicon reads from V6 16S rRNA region and 430,403 WGS reads generated using the 454 platform. Similar strategies for taxonomic and functional assignment were employed for all datasets. The microbial community structure of YAM (YN and YJ) was compared with Freshwater (FA and FN) and Sewage (SW) datasets. Bray-Curtis distances among the samples were calculated using taxonomic and functional (KEGG and EggNOG) profile [62] and analyzed using Principal Coordinate Analysis (PCoA). A comparison in the abundance of Antibiotic Resistance Genes (ARGs) identified using CARD database was also carried out for the five datasets.

### Data availability

The nucleotide paired-end sequences generated in this study have been deposited in NCBI under the BioProject ID PRJNA531627, and can accessed using the NCBI SRA accession id SRR8870486, SRR8870487, SRR8870488 and SRR8870489.

## Supplementary information

Additional file 1: **Table S1** Raw and high quality reads in the two Yamuna samples and other datasets. **Table S2** Distribution of top ten OTUs annotated as Aeromonadaceae in YN and their blast hits summary. **Table S3** Distribution of top ten OTUs in YJ and their blast hits summary. **Figure S1** Distribution of family showing more than 1% abundance in the two YM datasets. **Figure S2** Alpha diversity of microbes in the five datasets. (A) Number of OTUs. (B) Shannon index. **Figure S3** PCoA plot showing the Bray-curtis distance among the five datasets based on eggNOG categories. **Figure S4** Comparison of Phylum showing more than 1% abundance in the five datasets. **Figure S5** Comparison of Family showing more than 1% abundance in the five datasets. (DOCX 471 kb)

Additional file 2: **Table S4.** Abundance of KEGG Pathways in the Yamuna. **Table S5.** Abundance of KEGG Orthologs in the Yamuna. **Table S6.** The antibiotic resistant genes identified in the Yamuna samples a) In YJ b) In YN. **Table S7.** Category wise Abundance of Antibiotic Resistant Genes. **Table S8.** List of metal resistance/tolerant genes identified in Yamuna and its count. **Table S9.** Abundance of KEGG Pathways in the five datasets. (XLSX 780 kb)

## Abbreviations

ARG: Antibiotic resistance genes
CARD: Comprehensive Antibiotic Resistance Database
CPCB: Central Pollution Control Board
FA: Freshwater August sample
FN: Freshwater November sample
MCP: Methyl-accepting chemotaxis protein
OTU: Operational taxonomic unit
SW: Sewage sample
YARG: Yamuna antibiotic resistance genes
YJ: Yamuna June sample
YN: Yamuna November sample
