# Supplementary material for: Metagenome of a polluted river reveals a reservoir of metabolic and antibiotic resistance genes
Source: Environ Microbiome. 2019 Sep 18;14:5. doi: 10.1186/s40793-019-0345-3 (PMC7989817; doi:10.1186/s40793-019-0345-3)
Supplement: Supplementary file 1 — Table S1 Raw and high quality reads in the two Yamuna samples and other datasets. Table S2 Distribution of top ten OTUs annotated as Aeromonadaceae in YN and their blast hits summary. Table S3 Distribution of top ten OTUs in YJ and their blast hits summary. Figure S1 Distribution of family showing more than 1% abundance in the two YM datasets. Figure S2 Alpha diversity of microbes in the five datasets. (A) Number of OTUs. (B) Shannon index. Figure S3 PCoA plot showing the Bray-curtis distance among the five datasets based on eggNOG categories. Figure S4 Comparison of Phylum showing more than 1% abundance in the five datasets. Figure S5 Comparison of Family showing more than 1% abundance in the five datasets. (DOCX 471 kb) [file 40793_2019_345_MOESM1_ESM.docx]

**Title**: Metagenome of a polluted river reveals a reservoir of metabolic and antibiotic resistance genes

**Authors:** Parul Mittal^1^, Vishnu Prasoodanan PK^1^, Darshan B. Dhakan^1^, Sanjiv Kumar^1,2^, Vineet K. Sharma^1^*

*: Corresponding author

**Affiliation:**

**^1^**Metagenomics and Systems Biology Group, Department of Biological Sciences, Indian Institute of Science Education and Research Bhopal, India.

**^2^**Division of Glycoscience, School of Biotechnology, Albanova University Center, Royal Institute of Technology, 10691 Stockholm, Sweden

*Corresponding author email:

vineetks@iiserb.ac.in

Email address of authors:

Parul Mittal: parulm@iiserb.ac.in; Vishnu Prasoodanan PK: vishnup16@iiserb.ac.in; Darshan B. Dhakan: darshan@iiserb.ac.in; Sanjiv Kumar: drsanjivk@gmail.com; Vineet K. Sharma: vineetks@iiserb.ac.in

**Supplementary Tables**

**Table S1** Raw and high quality reads in the two Yamuna samples and other datasets

|  |  | **YJ** | **YN** | **FA** | **FN** | **Sewage** |
| --- | --- | --- | --- | --- | --- | --- |
| Amplicon | Raw Reads | 13,565,755 | 191,740,397 | 235,469 | 256,503 | 237,559 |
|  | HQ Reads | 7,451,906 | 7,001,088 | 235,271 | 255,189 | 231,470 |
| Metagenome | Raw Reads | 96,000,349 | 165,873,760 | 61,659,612 | 34,204,450 | 430,403 |
|  | HQ Reads | 25,877,683 | 87,301,705 | 24,830,869 | 5,789,410 | 428,456 |

**Table S2** Distribution of top ten OTUs annotated as Aeromonadaceae in YN and their blast hits summary

| **OTUID** | **Blast hit** | **Query Length** | **OTU size** | **Abundance** | **Identity** | **Coverage** |
| --- | --- | --- | --- | --- | --- | --- |
| denovo48440 | Aeromonas hydrophila strain | 159 | 16575 | 0.0139396 | 97.00% | 100.00% |
|  | Aeromonas caviae strain |  |  |  |  |  |
| denovo324930 | Aeromonas hydrophila strain | 159 | 22910 | 0.0192674 | 96.00% | 100.00% |
|  | Aeromonas caviae strain |  |  |  |  |  |
| denovo320238 | Aeromonas hydrophila strain | 159 | 87441 | 0.0735380 | 97.00% | 100.00% |
|  | Aeromonas caviae strain |  |  |  |  |  |
| denovo315643 | Uncultured Aeromonas sp. clone BSB6-2 | 159 | 30528 | 0.0256741 | 97.00% | 100.00% |
|  | Aeromonas hydrophila strain |  |  |  |  |  |
|  | Aeromonas caviae strain |  |  |  |  |  |
| denovo277999 | Aeromonas hydrophila strain | 159 | 11961 | 0.0100592 | 97.00% | 100.00% |
|  | Aeromonas caviae strain |  |  |  |  |  |
| denovo225778 | Aeromonas hydrophila 4AK4, complete genome | 112 | 13613 | 0.0114484 | 91.00% | 97.00% |
| denovo188965 | Aeromonas hydrophila strain | 165 | 125910 | 0.1058905 | 96.00% | 100.00% |
|  | Aeromonas caviae strain |  |  |  |  |  |
| denovo1693 | Aeromonas hydrophila strain | 165 | 10690 | 0.0089903 | 95.00% | 100.00% |
|  | Aeromonas caviae strain |  |  |  |  |  |
| 839376 | Aeromonas hydrophila strain | 159 | 341765 | 0.2874250 | 98.00% | 100.00% |
|  | Aeromonas caviae strain |  |  |  |  |  |
| 839235 | Aeromonas hydrophila | 159 | 21247 | 0.0178688 | 97.00% | 100.00% |
|  | Aeromonas caviae strain |  |  |  |  |  |
|  | Aeromonas dhakensis strain |  |  |  |  |  |

**Table S3** Distribution of top ten OTUs in YJ and their blast hits summary

| **OTUID** | **Blast hit** | **Query Length** | **OTU size** | **Abundance** | **Identity** | **Coverage** |
| --- | --- | --- | --- | --- | --- | --- |
| 1104386 | Uncultured bacterium clone DWTP1.1B.E09 16S ribosomal RNA gene, partial sequence | 154 | 127447 | 0.0185528 | 100.00% | 100.00% |
|  | Cloacibacterium normanense strain YL 16S ribosomal RNA gene, partial sequence |  |  |  |  |  |
|  | Uncultured Flavobacteriia bacterium clone XW13 16S ribosomal RNA gene, partial sequence |  |  |  |  |  |
|  | Uncultured Bergeyella sp. clone BL-113995 16S ribosomal RNA gene, partial sequence |  |  |  |  |  |
| 1106652 | Acinetobacter lwoffii strain JP4 16S ribosomal RNA gene, partial sequence | 160 | 402428 | 0.0585824 | 100.00% | 100.00% |
|  | Acinetobacter variabilis strain NIPH 2171 16S ribosomal RNA, complete sequence |  |  |  |  |  |
|  | Acinetobacter sp. RKEM 532 16S ribosomal RNA gene, partial sequence |  |  |  |  |  |
|  | Acinetobacter sp. I2 16S ribosomal RNA gene, partial sequence |  |  |  |  |  |
|  | Acinetobacter indicus strain 273 16S ribosomal RNA gene, partial sequence |  |  |  |  |  |
|  | Acinetobacter sp. 0051KARWAR 16S ribosomal RNA gene, partial sequence |  |  |  |  |  |
| 1107256 | Aeromonas hydrophila | 159 | 420524 | 0.0612167 | 100.00% | 100.00% |
|  | Aeromonas caviae |  |  |  |  |  |
|  | Aeromonas enteropelogenes |  |  |  |  |  |
|  | Aeromonas jandaei strain |  |  |  |  |  |
| 1110959 | Acinetobacter tjernbergiae | 160 | 283855 | 0.0413215 | 100.00% | 100.00% |
|  | Acinetobacter sp. CC |  |  |  |  |  |
|  | Acinetobacter sp. 89B2 |  |  |  |  |  |
|  | Acinetobacter junii strain A2 |  |  |  |  |  |
| 1111801 | Aeromonas veronii | 159 | 115804 | 0.0168579 | 100.00% | 100.00% |
|  | Aeromonas salmonicida |  |  |  |  |  |
| 1111886 | Novosphingobium sp. D39 | 134 | 270488 | 0.0393756 | 100.00% | 100.00% |
|  | Novosphingobium aquiterrae |  |  |  |  |  |
|  | Novosphingobium sp. N002N |  |  |  |  |  |
|  | Novosphingobium sp. HME8524 |  |  |  |  |  |
|  | Novosphingobium sp. Dbr-01 |  |  |  |  |  |
| 831485 | Flavobacterium cucumis strain | 154 | 589800 | 0.0858586 | 100.00% | 100.00% |
|  | Uncultured bacterium clone ISSEP-73 16S ribosomal RNA gene, partial sequence |  |  |  |  |  |
|  | Uncultured bacterium clone WB40 16S ribosomal RNA gene, partial sequence |  |  |  |  |  |
|  | Uncultured bacterium clone CB-20 16S ribosomal RNA gene, partial sequence |  |  |  |  |  |
|  | Uncultured bacterium clone CB-4 16S ribosomal RNA gene, partial sequence |  |  |  |  |  |
|  | Uncultured bacterium gene for 16S ribosomal RNA, partial sequence, clone: FLB-6 |  |  |  |  |  |
|  | Uncultured bacterium clone T6_1110_80 16S ribosomal RNA gene, partial sequence |  |  |  |  |  |
|  | Uncultured bacterium clone J304-42 16S ribosomal RNA gene, partial sequence |  |  |  |  |  |
|  | Activated sludge bacterium OC13 gene for 16S ribosomal RNA, partial sequence |  |  |  |  |  |
|  | Bacterium 182_oclvp528 16S ribosomal RNA gene, partial sequence |  |  |  |  |  |
|  | Bacterium 181_oclvp548 16S ribosomal RNA gene, partial sequence |  |  |  |  |  |
| denovo283506 | Acinetobacter sp. 815B5_12ER2A | 160 | 195590 | 0.0284725 | 100.00% | 100.00% |
|  | Acinetobacter towneri |  |  |  |  |  |
|  | Acinetobacter soli strain |  |  |  |  |  |
|  | Acinetobacter sp. NF404 |  |  |  |  |  |
| denovo308175 | Acinetobacter lwoffii | 160 | 159061 | 0.0231549 | 99.00% | 100.00% |
|  | Acinetobacter variabilis |  |  |  |  |  |
|  | Acinetobacter seohaensis |  |  |  |  |  |
|  | Acinetobacter sp. RKEM 532 |  |  |  |  |  |
|  | Uncultured bacterium clone I3Q1XXJ02B3K41 16S ribosomal RNA gene, partial sequence |  |  |  |  |  |
|  | Uncultured bacterium clone HF43 16S ribosomal RNA gene, partial sequence |  |  |  |  |  |
|  | Uncultured bacterium clone B176_442 small subunit ribosomal RNA gene, partial sequence |  |  |  |  |  |
|  | Uncultured Alkanindiges sp. clone DVBSW_J358 16S ribosomal RNA gene, partial sequence |  |  |  |  |  |
|  | Uncultured bacterium clone B9-70 16S ribosomal RNA gene, partial sequence |  |  |  |  |  |
|  | Uncultured bacterium clone ncd2574d07c1 16S ribosomal RNA gene, partial sequence |  |  |  |  |  |
| 1109303 | Acinetobacter tjernbergiae strain | 160 | 266766 | 0.0388338 | 100.00% | 100.00% |
|  | Acinetobacter sp. CC 141109 |  |  |  |  |  |
|  | Acinetobacter sp. CC 141104 |  |  |  |  |  |
|  | Acinetobacter sp. CC 141103 |  |  |  |  |  |
|  | Acinetobacter sp. 90A |  |  |  |  |  |

**Supplementary Figures**

**
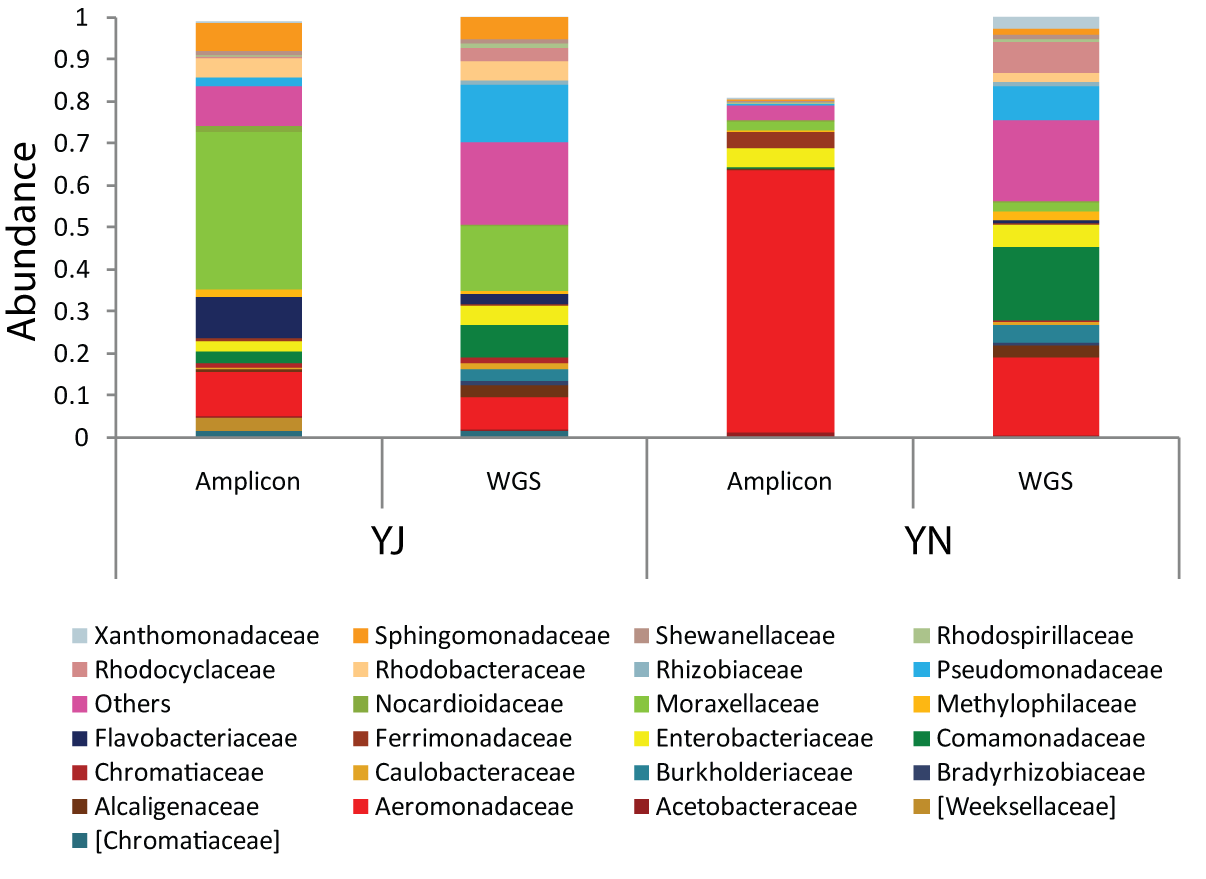
**

**Figure S1** Distribution of family showing more than 1% abundance in the two YM datasets

**
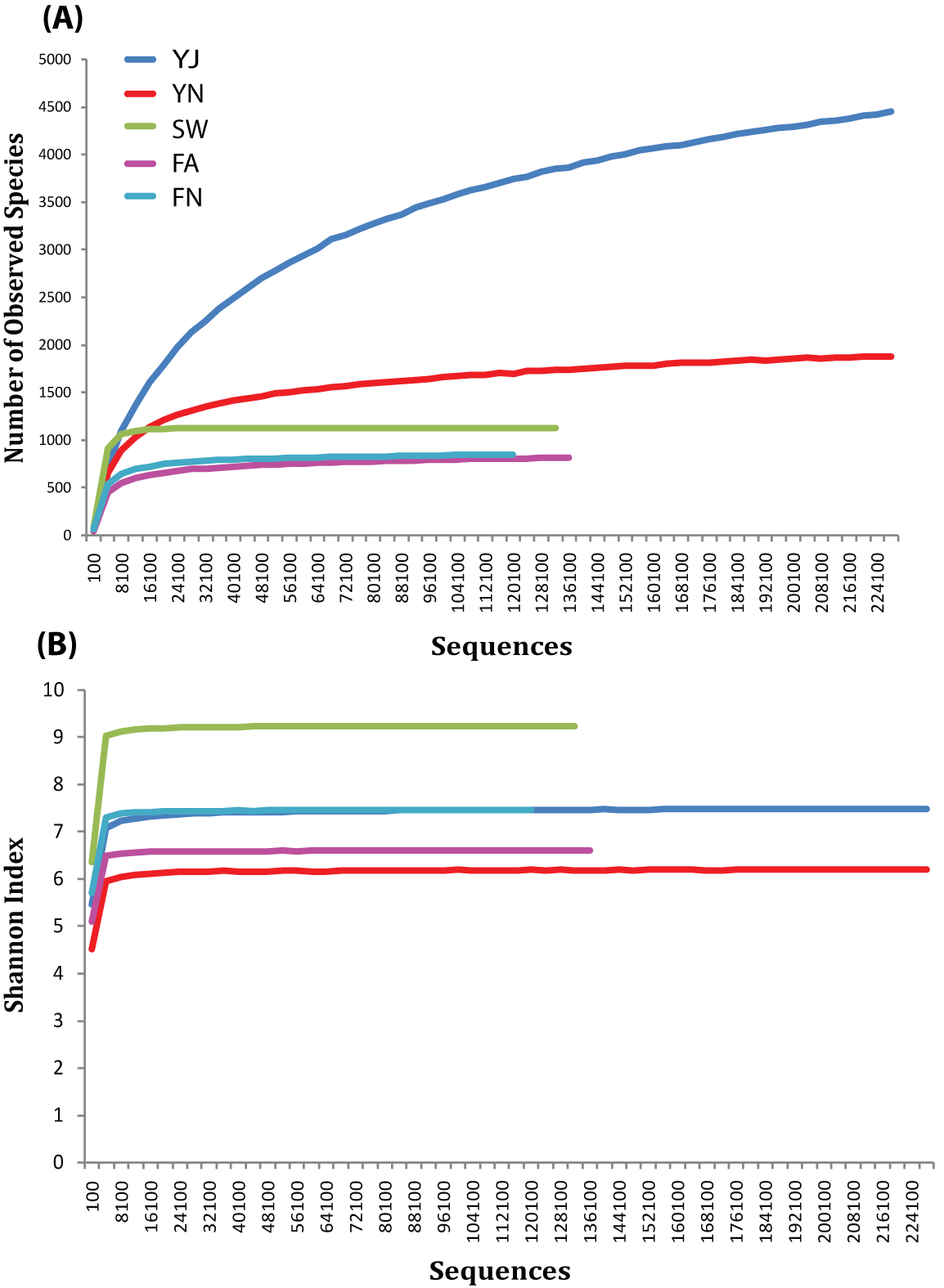
**

**Figure S2** Alpha diversity of microbes in the five datasets. **(A)** Number of OTUs. **(B)** Shannon index.

**
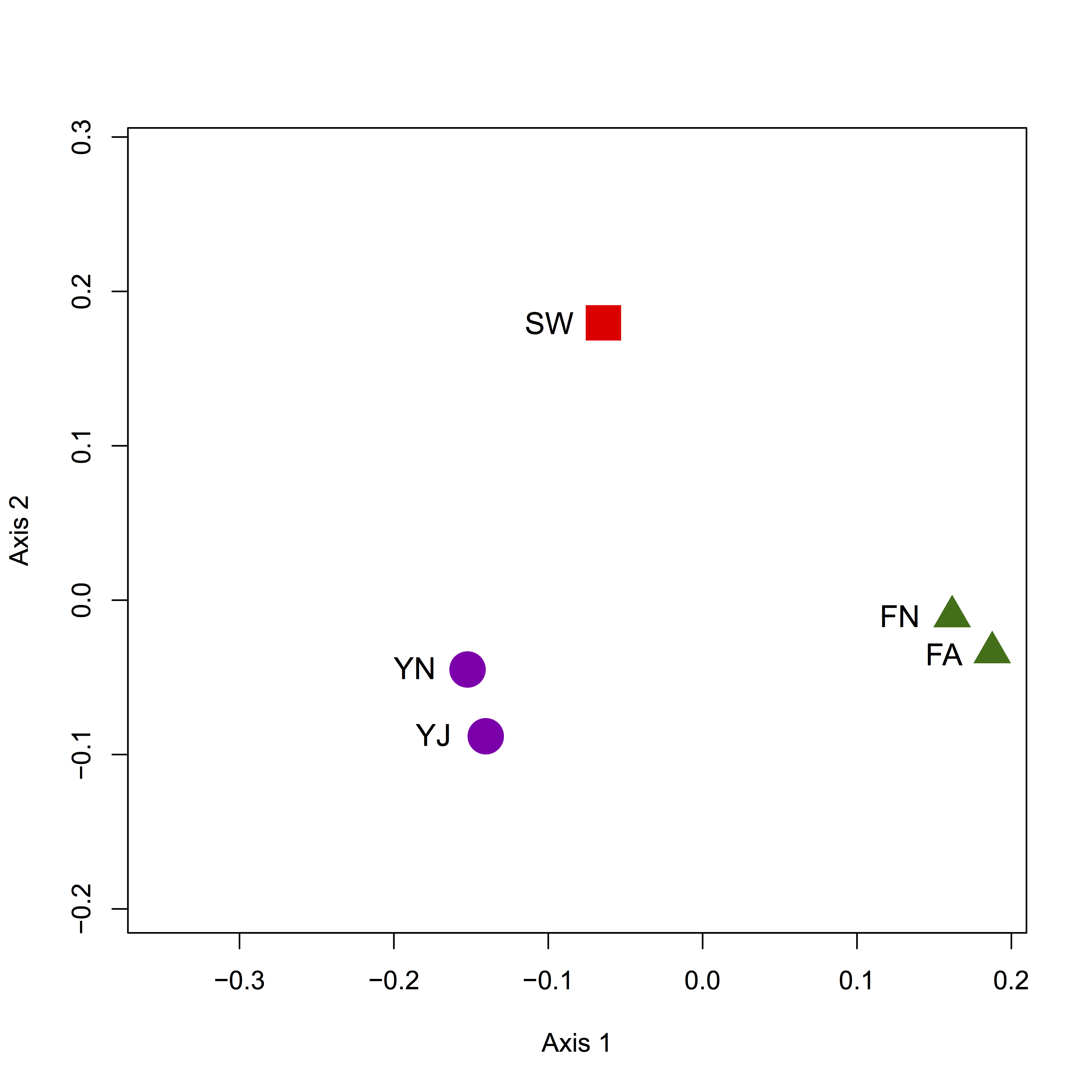
**

**Figure S3** PCoA plot showing the Bray-curtis distance among the five datasets based on eggNOG categories

**
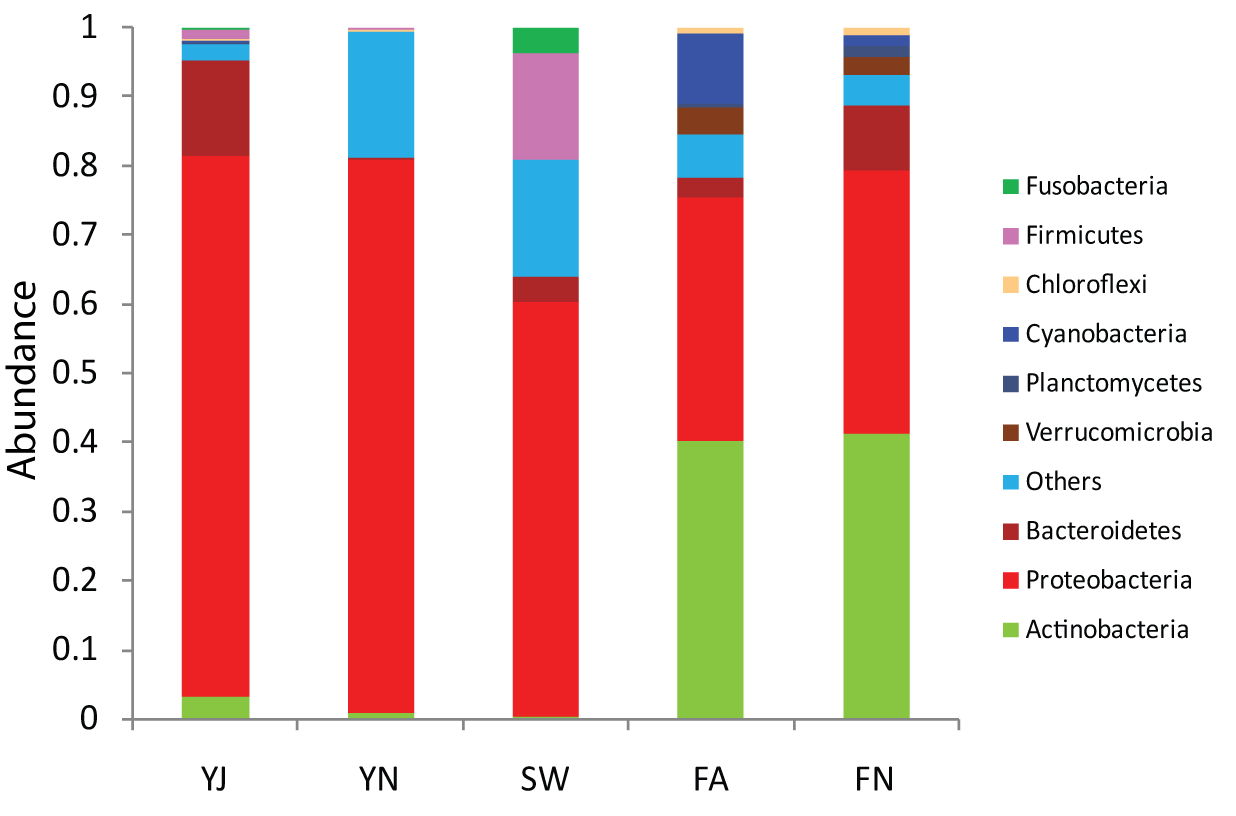
**

**Figure S4** Comparison of Phylum showing more than 1% abundance in the five datasets.

**
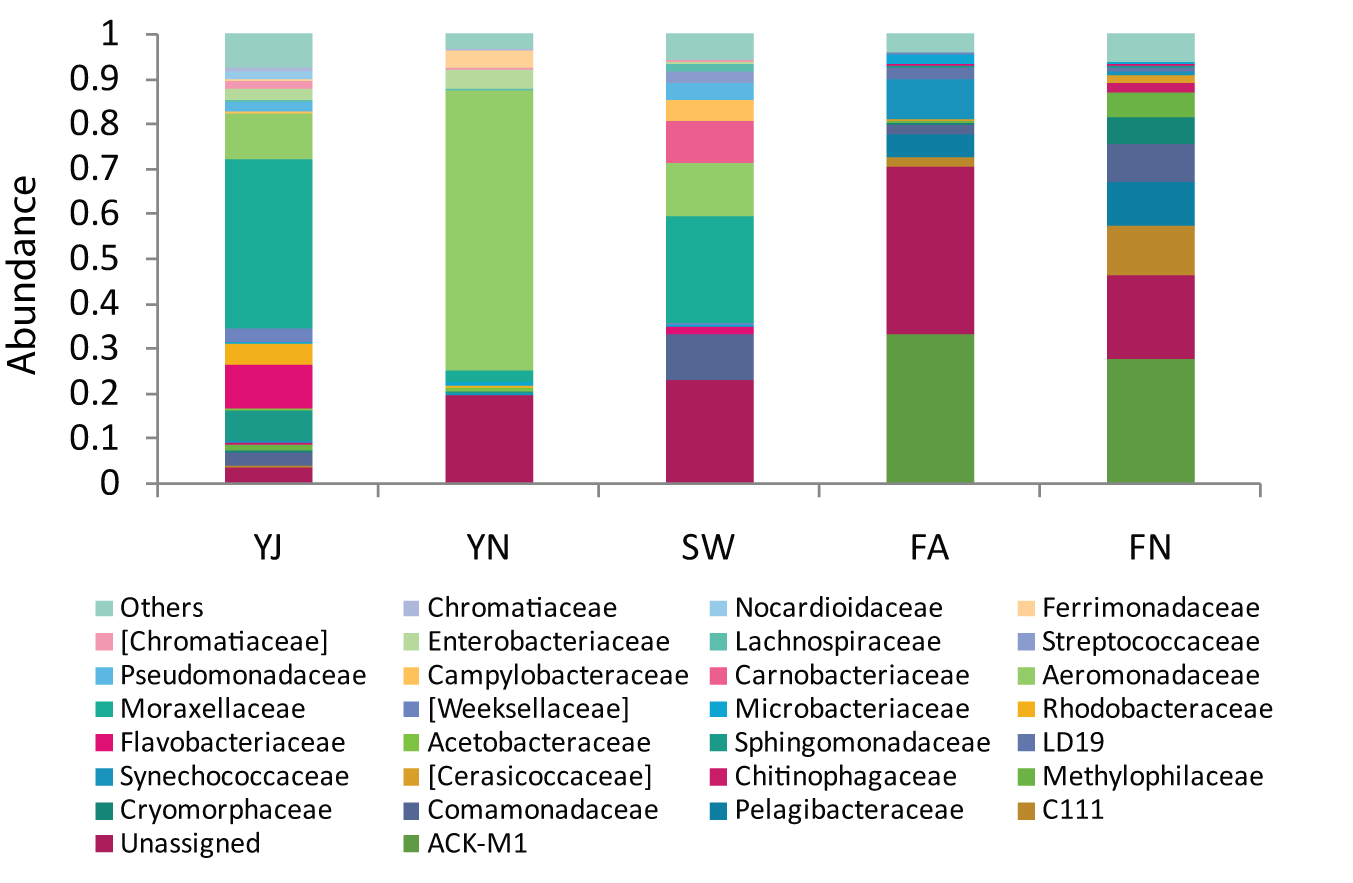
**

**Figure S5** Comparison of Family showing more than 1% abundance in the five datasets.
